# Endothelial Cells Potentially Participate in the Metastasis of Triple-Negative Breast Cancer

**DOI:** 10.1155/2022/5412007

**Published:** 2022-02-27

**Authors:** Yanfei Ma, Yanghong Li, Pengwei Guo, Jingjie Zhao, Qiang Qin, Jiajia Wang, Zhengfang Liang, Dalong Wei, Zechen Wang, Jiajia Shen, Siyuan He, Qianli Tang, Guanming Lu, Guiling Shi, Lingzhang Meng

**Affiliations:** ^1^Jinan University, Guangzhou, Guangdong Province, China; ^2^Center for Systemic Inflammation Research (CSIR), Youjiang Medical University for Nationalities, Baise, Guangxi Province, China; ^3^Department of Gland Surgery, Affiliated Hospital of Youjiang Medical University for Nationalities, Baise, Guangxi Province, China; ^4^Department of Renal Diseases, Affiliated Hospital of Youjiang Medical University for Nationalities, Baise, Guangxi Province, China; ^5^Life Science and Clinical Research Center, Affiliated Hospital of Youjiang Medical University for Nationalities, Baise, China; ^6^Department of Gland Surgery, Nanning Maternity and Child Health Hospital, Nanning, Guangxi Province, China; ^7^Department of Obstetrics and Gynecology, Affiliated Hospital of Youjiang Medical University for Nationalities, Baise City, Guangxi Province, China; ^8^Department of Urinary Surgery, Affiliated Hospital of Youjiang Medical University for Nationalities, Baise, Guangxi Province, China; ^9^Burn Plastic & Trauma Surgery Department, Affiliated Hospital of Youjiang Medical University for Nationalities, Baise, Guangxi Province, China

## Abstract

Inhibition of triple-negative breast cancer metastasis has long been a challenge, mainly due to the difficulty in identifying factors that contribute to this process. In this study, freshly isolated triple-negative breast cancer biopsied cells obtained from consenting patients were subjected to flow cytometry and bioinformatic analysis to identify three endothelial cell subclusters: EC (*ATP1B3*), EC (*HSPA1B*), and EC (*KRT7*) in the tumor microenvironment. These endothelial cell subclusters exhibited distinguishing biological features. Based on differentially expressed genes derived from the subclusters, gene set enrichment analysis showed that EC (*ATP1B3*) and EC (*HSPA1B*) contribute to the process of metastasis, for example, in fibrosarcoma and anaplastic carcinoma. In this study, we identified the heterogeneity of endothelial cells in the human breast cancer and have provided insights into its role in metastasis.

## 1. Introduction

Triple-negative breast cancer (TNBC) is characterized by low or no expression of the progesterone, oestrogen, and human epidermal growth factor 2 receptors [1]. Approximately 20% of the breast cancer patients are diagnosed with TNBC [2, 3]. Current therapies against TNBC include surgery, chemotherapy, and/or immune therapy, and although this has led to decreased mortality, numerous patients still undergo cancer metastasis to other organs, such as the brain, lung, and bone [2–4], resulting in a drastic loss of therapeutic efficiency and quality of life. The processes underlying TNBC metastasis remain unclear due, in part, to the complexity of this process [3].

The tumor microenvironment (TME) participates in tumor cell proliferation, apoptosis, and migration [5], all of which are closely related to metastasis. The cellular components in the TME include monocytes, macrophages, neutrophils, B cells, and endothelial cells [6–8]. Monocytes facilitate the breast tumor metastasis by CCL2-induced recruitments [9], and macrophages bridge the tumor cell-extracellular matrix towards metastasis by secreting SPARC [10]. Neutrophils promote metastasis by forming extracellular traps [11, 12] and B-lineage cells contribute to metastasis by upregulating STX16 and ATIC [7]. Although endothelial cells have long been considered a key cell type in tumor initiation and progression [13], their role in TNBC metastasis has yet to be fully elucidated.

The endothelial cells are heterogenous in both healthy and pathological conditions [14–17].Exploring the heterogeneity of endothelial cells in TNBC would help to understand their role in metastasis. In this study, three endothelial cell subpopulations were identified from TNBC biopsies, two of which could contribute to metastasis to fibrosarcoma and anaplastic carcinoma.

## 2. Materials and Methods

### 2.1. Human Biopsies

Biopsies were isolated from TNBC patients underwent surgery. After pathological examination, the residual biopsies were processed either for flow cytometry analysis or immunofluorescent staining. This study was approved by the Ethics Committee of Youjiang Medical University for Nationalities.

### 2.2. Flow Cytometry Analysis

Freshly isolated TNBC biopsies were digested with collagenase IV (40 mg/mL, Gibco, #17104-019) and filtered through a 100 *μ*m stainless strainer to obtain single-cell suspensions. The cells were resuspended in PBS containing 0.5% BSA and 2 mM EDTA. The prepared single-cell suspensions were blocked with an Fc*γ* receptor blocker and then incubated with fluorescence-labelled antibodies on ice for 15 min for surface staining. After washing twice with PBS, the cells were fixed and permeabilised with a Cytofix/Cytoperm Kit (BD, #554717) and then incubated with fluorescence-labelled antibodies for intracellular staining. After washing twice with PBS containing 0.5% BSA, the cells were resuspended in PBS containing 0.5% BSA and 2 mM EDTA. A flow cytometer (Thermo Fisher Attune NxT) was used to measure the events. The antibodies used in this study included PE anti-CD31 (Biolegend, #303106), Fixable Viability Dye–eFluor 450 (Biolegend, #65-0863-14), 7-AAD (Invitrogen, #A1310), APC anti-human CD298/ATP1B3 (Biolegend, #341706), FITC anti-HSPA1B (Cusabio, #CSB-PA28047C0Rb), Alexa Fluor anti-KRT7 (Novus, #NBP2-47944 AF700), APC anti-CD93 (Biolegend, #336120), and FITC anti-CDH5 (LSBio, #LS-C467144-100).

### 2.3. Immunofluorescent Microscopy

Biopsies were embedded in 10 × 10 × 5 mm mold (Sakura, #4565) and then sectioned to 4 *μ*m thickness with microtome (LEICA CM1950). After fixation with −20°C methanol, the slices were washed twice with PBS. Then incubated with primary antibodies overnight at 4°C, after washing 3 times with PBS, slices were incubated with fluorescent-labelled secondary antibodies. After washing twice with PBS, sections were mounted with Fluoromount-G (SouthernBiotech, #0100-01). The images were captured by immunofluorescent microscope (LEICA DMI3000B). The primary antibodies used in this study include the following: mouse-anti-human CD31 (Invitrogen, #14-0311-85), rabbit-anti-human ATP1B3 (Invitrogen, #PA5-119425), rabbit-anti-human HSPA1B (Invitrogen, # PA5-28369), and rabbit-anti-human KRT7 (Invitrogen, #MA1-06316). The secondary antibodies used in this study include the following: A555 goat-anti-mouse IgG1 (Invitrogen, #A21127) and A488 donkey-anti-rabbit IgG (Invitrogen, #A21206).

### 2.4. scRNA-Seq Bioinformatics Analysis

The scRAN-seq data of TNBC and control biopsied tissues were retrieved from the NCBI GEO database (https://www.ncbi.nlm.nih.gov/geo/) under the accession code GSE161529 [18]. In total, four TNBC tissues and 13 normal tissues were obtained. Cells were clustered with R package Seurat (v4.0.2) at a resolution of 0.1, and differentially expressed genes (DEGs) were evaluated and processed using the R package EnhancedVolcano (v1.11.3). Gene set enrichment analysis (GSEA) was performed using the R package clusterProfiler (v4.0.0).

## 3. Results

### 3.1. Identification of Endothelial Cells in the Human Breast and TNBC Biopsied Tissues

**Figure 1:**
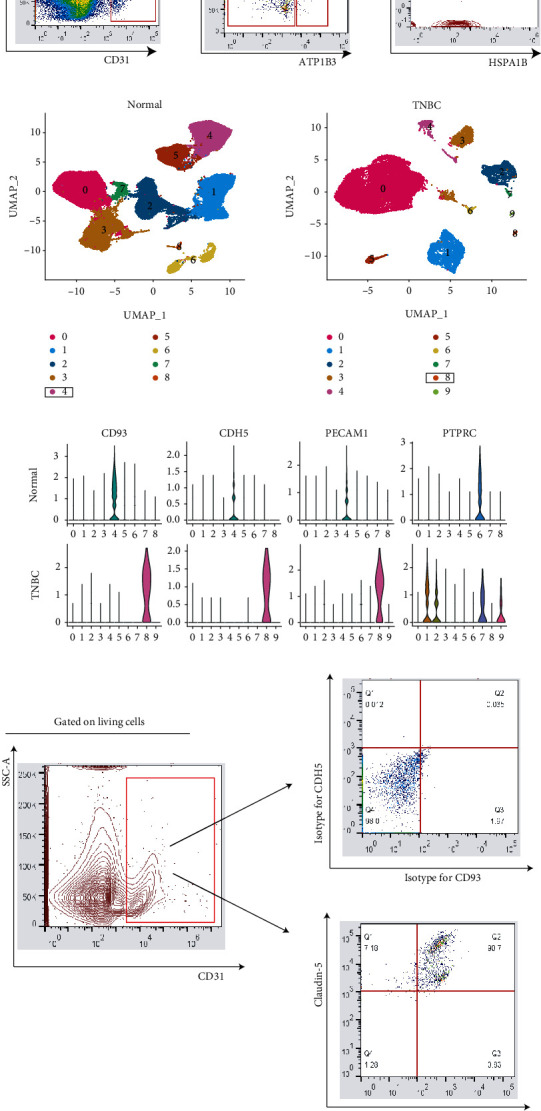
Identification of endothelial cells in TNBC biopsies. (a) Flow cytometry analysis revealed three endothelial cell subclusters in TNBC biopsied tissues, featured by the expression of ATP1B3, HSPA1B, and KRT7, respectively. Data represent similar results acquired from five independent experiments. (b) UMAP plots showed 9 cell types in the normal human breast and 10 cell types in TNBC biopsy tissues. The boxed cell types indicate endothelial cells. (c) Violin plots exhibited the expression pattern of endothelial cell markers (CD93, CDH5, and PECAM1) and a leukocyte marker (PTPRC). (d) Flow cytometry analysis identified the expression of CDH5 and CD93 from endothelial cells in TNBC biopsies. Data represent similar results acquired from three independent experiments.

In the freshly isolated malignant cells from patients with TNBC, a significant proportion of endothelial cells (CD31^+^) were detected (Figure 1(a)). To detect whether they could be divided into subpopulations, the expression of ATP1B [19], HSPA1B [20], and KRT7 [21] were evaluated. Interestingly, TNBC endothelial cells could be further divided into three subpopulations: EC (ATP1B3), EC (HSPA1B), and EC (KRT7) (Figure 1(a)). To compare their genetic profiles, the scRNA-Seq data of normal human breast tissues and TNBC tissues were retrieved [18]. Overall, nine cell types were identified in normal tissues and 10 in TNBC tissues (Figure 1(b)). Three markers, CD93, CDH5 (coding), and PECAM1, were used to isolate endothelial cells (Figure 1(c), Supplementary Figure 1) [22]. The fifth population in normal breast tissue and the ninth population in TNBC tissues were endothelial cells (Figures 1(b) and 1(c)). The lack of expression of PTPRC (encoding CD45) further suggested that these two populations did not belong to leukocytes (Figure 1(c), Supplementary Figure 1). Flow cytometry analysis showed that TNBC endothelial cells constitutively expressed CDH5 and CD93 (Figure 1(d)).

### 3.2. The Human Breast and TNBC Endothelial Cells Are Heterogeneous

**Figure 2:**
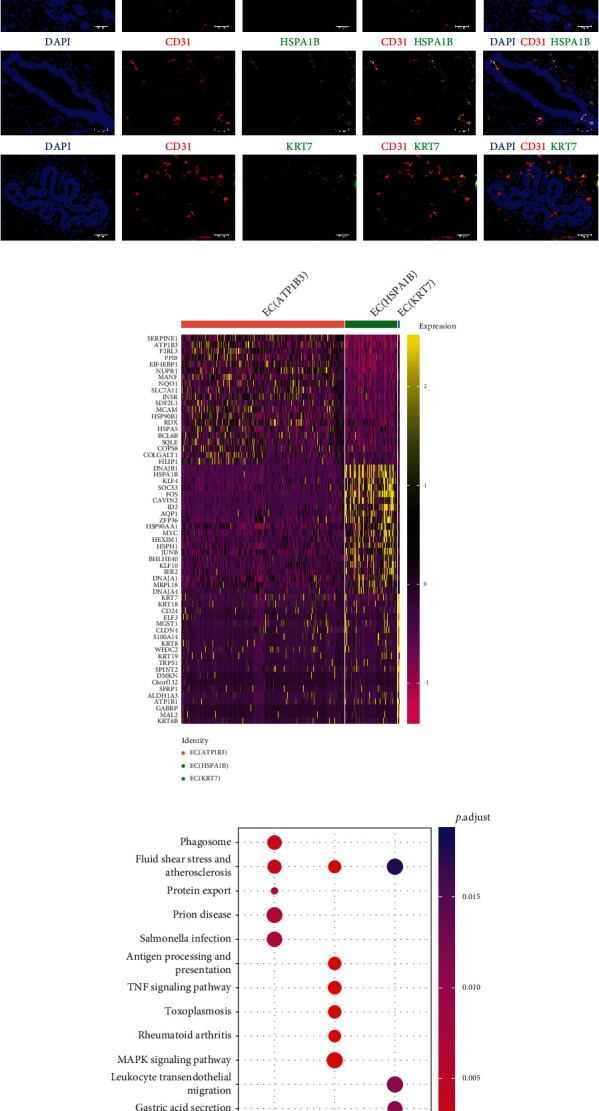
scRNA-seq analysis showed three subclusters in breast-associated biopsies. (a) UMAP plot showed integration of endothelial cells isolated from normal breast and TNBC tissues. Three subclusters were identified based on genetic profiles. (b) Violin plots showed the expression pattern of three feature genes *ATP1B3*, *HSPA1B*, and *KRT7.* (c) Immunofluorescent staining of 3 endothelial cell subpopulations: EC (ATP1B3), EC (HSPA1B), and EC (KRT7). Endothelial cells were labelled with CD31. (d) Heatmap showed expression pattern of top 20 genes from each endothelial cell subclusters. (e) Dot plot revealed that each endothelial cell subcluster possesses distinct biological features.

To compare the biological features of endothelial cell subpopulations, isolated endothelial cells from the scRNAN-seq experiment were evaluated. The integration of the human breast and TNBC endothelial cells revealed three subclusters (Figure 2(a)). Interestingly, ATP1B3, HSPA1B, and KRT7 were preferentially expressed in three endothelial cell subpopulations (Figure 2(b)), which is consistent with the flow cytometry analysis (Figure 1(a)). To determine distribution pattern of these 3 endothelial cell subpopulations, we performed immunofluorescent staining. Obviously, these 3 subpopulations preferentially exist in different regions of TNBC biopsies, which could be indicated by DAPI staining (Figure 2(c)). In total, 292 genes were expressed at higher levels in EC (ATP1B3), 505 were higher in EC (HSAP1B), and 118 genes were expressed at higher levels in EC (KRT7), compared among these three subpopulations (Supplementary Figure 2). The heat map shows a brief view of the similarity/disparity of the top 20 genes from the three subpopulations (Figure 2(d)). Biological theme comparison analysis (Figure 2(e), Supplementary Figure 3) of these subpopulation indicates that EC (ATP1B3) plays a vital role in mediating phagocytosis and fluid shear stress and increases the susceptibility to develop an atherosclerosis, prion disease, and salmonella infection in the breast cancer patients [23–25]; EC (HSPA1B) could promote in antigen presentation and MAPK signalling and participates in mediating rheumatoid arthritis [26] and toxoplasmosis [27] in the breast cancer patients; and EC (KRT7) could be more important for mediating leukocyte transendothelial migration and thyroid hormone signalling. Interestingly, EC (ATP1B3) exhibited higher expression of phagocytosis-related genes in TNBC patients, RAB5A and EEA1 (Supplementary Figure 4), indicating these patients could be more susceptible to infection.

### 3.3. Endothelial Cell Subpopulations Contribute to TNBC Metastasis

**Figure 3:**
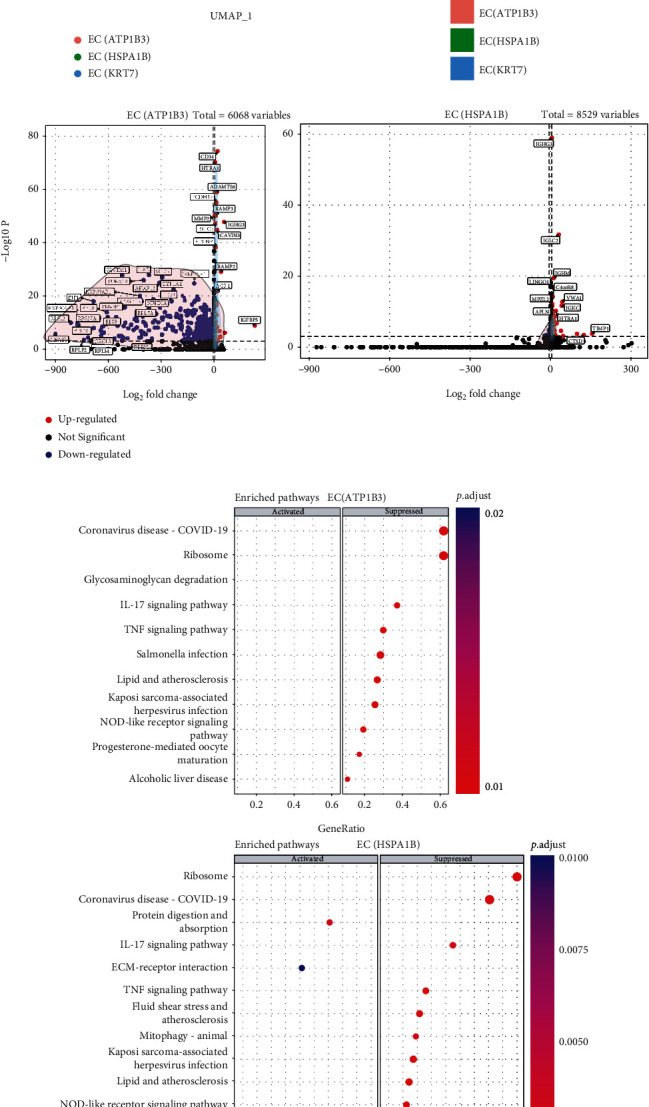
Comparison of TNBC endothelial cell subclusters with the normal human breast tissue. (a) Split UMAP plot showed the distribution of endothelial cell subclusters in both normal biopsies and TNBC biopsies. (b) Stacked bar plot showed a comparison of the frequency of endothelial cell subclusters, normal vs TNBC biopsies. (c) Volcano plots showed the distribution of DEGs calculated from endothelial cell subcluster EC (ATP1B3) and EC (HSPA1B). (d) Dot plots showed up and downregulated pathways in the endothelial cell subcluster EC (ATP1B3) and EC (HSPA1B).

In comparison to the normal human breast endothelial cell subpopulations, integrated scRNA-seq data revealed that the distribution and frequencies of these subpopulations were different (Figures 3(a) and 3(b)). The proportion of subpopulation EC (ATP1B3) increased significantly (Figure 3(b)), indicating that it is more important in the development of TNBC. Further calculation revealed 6068 DEGs in EC (ATP1B3) (TNBC vs. normal human breast) and 8529 DEGs in EC (HSPA1B) (Figure 3(c)). Unfortunately, the DEGs from EC (KRT7) could not be calculated because of their extremely low numbers. GSEA analysis revealed that most of the pathways were downregulated in TNBC endothelial cell subpopulations compared to the normal human breast tissues, such as IL-17 signalling (including *IL17RA*, *TRAF3IP2*, *GSK3B*, and *NFKB1*), TNF signalling (including *TNFR1*, *TRADD*, *TAB2*, and *RIP1*), and NOD-like receptor signalling (including *NFR1*, *NFR5*, and *NIN*) (Figure 3(d)), all of which are highly related to the development of cancer [28, 29]. Moreover, EC (HSPA1B) in the TNBC tissues exhibited altered fluid shear stress compared to the normal human breast tissues (Figure 3(d)), which participates in tumor metastasis [30].

**Figure 4:**
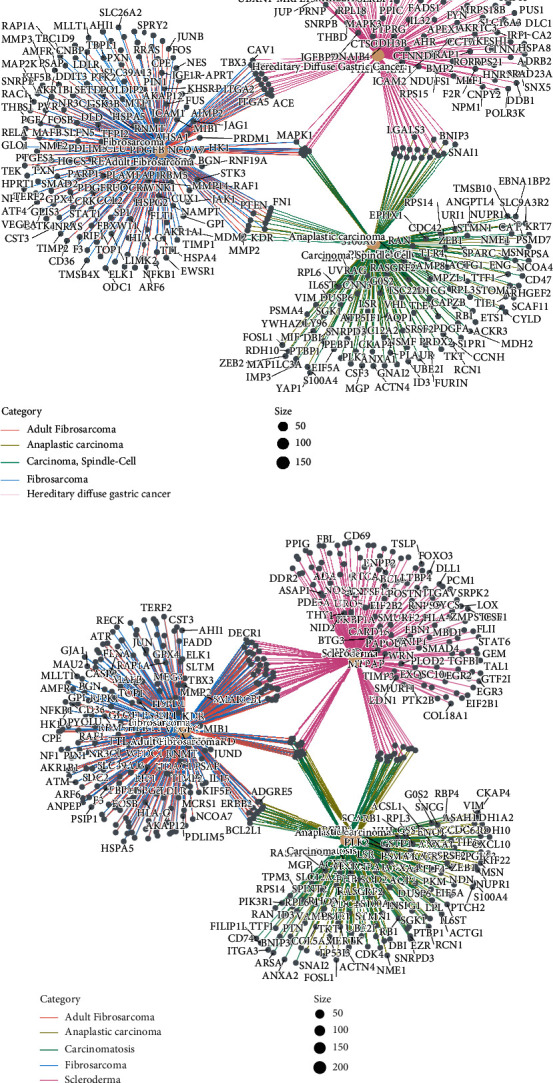
Endothelial cells in TNBC biopsies contribute to metastasis. (a) CNET plot shows the DEGS of EC (ATP1B3) and predicts metastasis. (b) CNET plot shows the DEGS of EC (HSPA1B) and predicts metastasis.

Gene concept network analysis of the DEGs abstracted from the above two endothelial cell subpopulations revealed that in TNBC, EC (ATP1B3) could contribute to TNBC metastasis to fibrosarcoma, hereditary diffuse gastric cancer, and anaplastic carcinoma (Figure 4(a)), while EC (HSPA1B) could participate in TMBC metastasis of fibrosarcoma and anaplastic carcinoma (Figure 4(b)).

## 4. Discussion

The lack of expression of progesterone, oestrogen, and human epidermal growth factor 2 receptors makes the current therapies against TNBC, such as chemotherapy, immunotherapy, less effective [3]. Numerous patients develop metastasis to distal organs [31]. TNBC patients are likely to develop fatal metastatic fibrosarcoma [3], gastric cancer [32], and anaplastic carcinoma [33]. Though it is known that cells and factors in the TME are key components driving TNBC metastasis, it remains challenging to identify such “foes”. For a long time, scientists have attempted to delineate immune cells, such as neutrophils, monocytes, macrophages, and B-lineage cells, in the process of TNBC metastasis; however, few studies have emphasised the role of endothelial cells in TNBCs. Recently, Huang et al. reported a correlation between fluid shear stress and tumor metastasis [30]. Rivoceranib treatment (targeting vascular endothelial growth factor-2) showed a prominent effect on metastatic gastric cancer, indicating that endothelial cells play a vital role in TNBC metastasis. However, the detailed cellular/molecular mechanisms still need to be elucidated. Besides, we discovered that endothelial cells in TNBC patients could respond abnormally to fluid shear stress (Figure 3(d)). It helps explain how cancer cells reverse transmigrate into nearby blood vessels and/or lymphatic vessels. Possibly, the cancer cells intravasate via two independent manners: through endothelial cell surface junctions and direct reverse transmigrate through endothelial cells. Inevitably, fluid shear stress could potentially facilitate the above procedures.

The advent of scRNA-seq makes it feasible to analyse endothelial cell genetic profiles [15, 16]. Using sequencing data, we first identified three endothelial cell subpopulations in the normal human breast and TNBC tissues and validated them by flow cytometry analysis. The heterogeneity of breast endothelial cells was studied with distinguished biological/pathological features of each subpopulation, and their genetic profiles were analysed. Moreover, the importance of endothelial cells in mediating TNBC metastasis has been delineated and discussed, especially the development of fibrosarcoma, hereditary diffuse gastric cancer, and anaplastic carcinoma in TNBC patients. Thus, endothelial cells could serve as a therapeutic target for TNBC metastasis.

However, the potential role of EC (KRT7) in TNBC metastasis has not been studied because of extreme cell events. Further studies should be conducted to bridge this gap. Moreover, TNBC metastasis should be evaluated with relevant genetic modified animal models in further studies.

## Data Availability

The datasets and code generated or analysed in this study are available from the corresponding author upon reasonable request.

## Supplementary Materials

Supplementary Materials Figure S1: feature plots exhibited the expression pattern of endothelial cell markers (CD93, CDH5, and PECAM1), and a leukocyte marker (PTPRC). Figure S2: genetic profile of EC subclusters (2). Figure S3: list of biocompare functional assay of EC subclusters (2). Figure S4: Expression of phagocytosis related genes in EC (ATP1B3) (2).
