# Supplementary figures and images for: Endothelial Cells Potentially Participate in the Metastasis of Triple-Negative Breast Cancer
Source: J Immunol Res. 2022 Feb 27;2022:5412007. doi: 10.1155/2022/5412007 (PMC8898858; doi:10.1155/2022/5412007)

Supplementary Figure 1

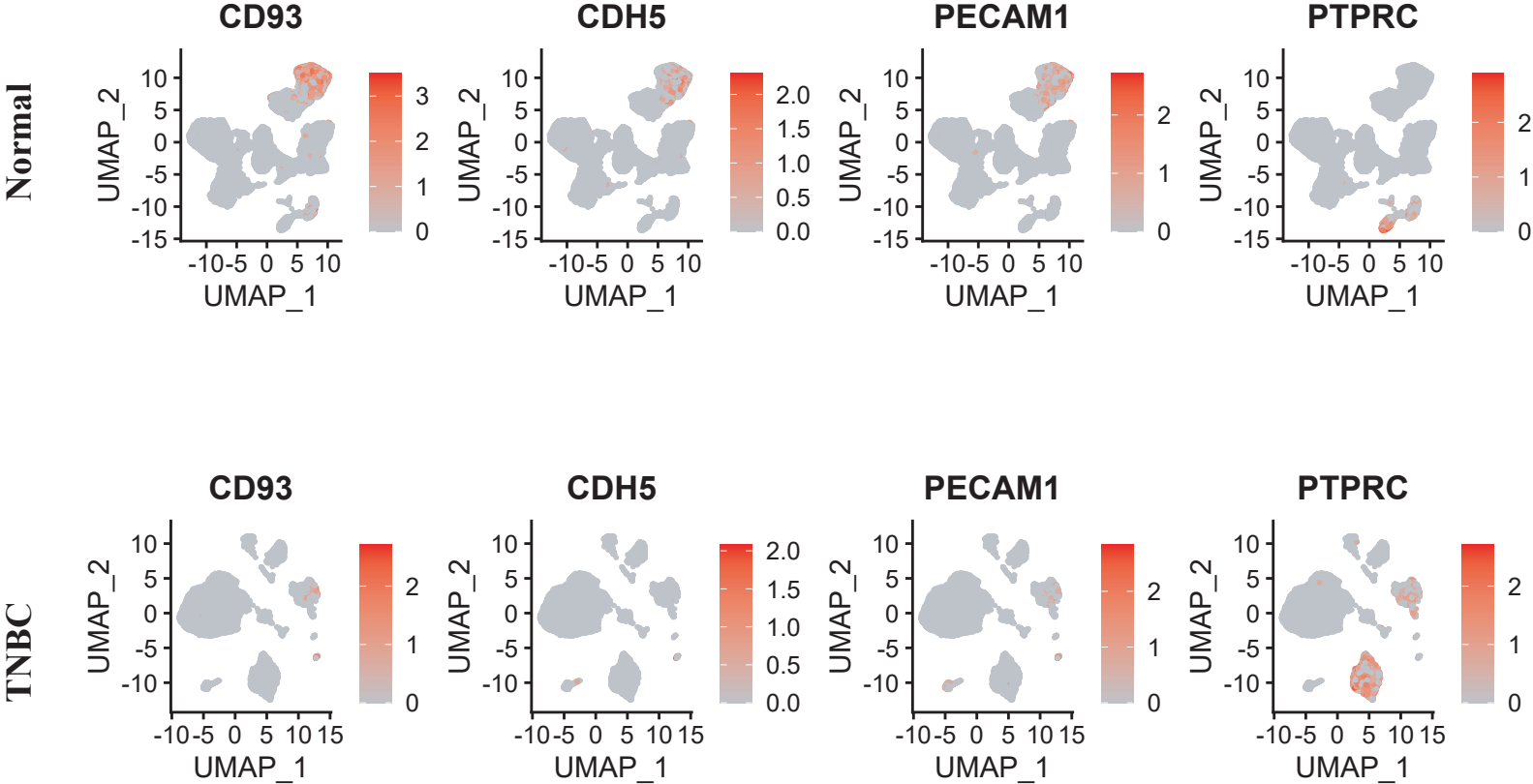

Supplement: Supplementary Materials — Figure S1: feature plots exhibited the expression pattern of endothelial cell markers (CD93, CDH5, and PECAM1), and a leukocyte marker (PTPRC). Figure S2: genetic profile of EC subclusters (2). Figure S3: list of biocompare functional assay of EC subclusters (2). Figure S4: Expression of phagocytosis related genes in EC (ATP1B3) (2). [file 5412007.f1.zip › Supplementary Figure 1. Feature plots exhibited the expression pattern of endothelial cell markers (CD93, CDH5, and PECAM1), and a leukocyte marker (PTPRC).pdf]

**Supplementary Figure 4**

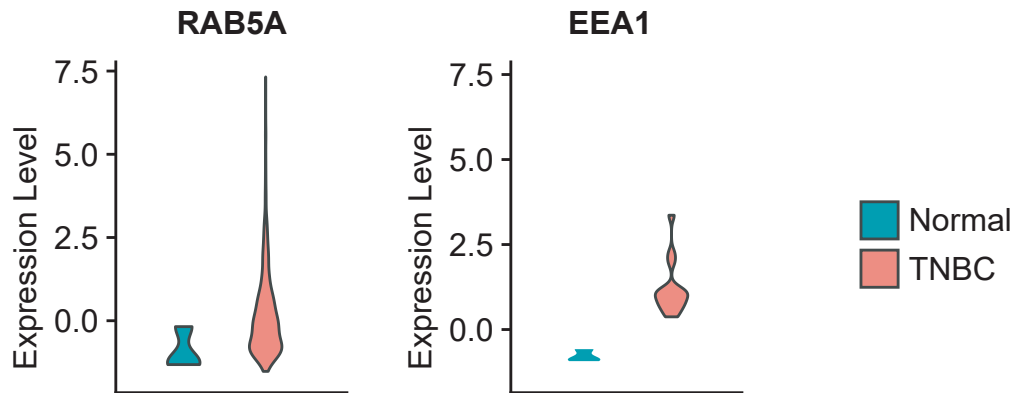

Supplement: Supplementary Materials — Figure S1: feature plots exhibited the expression pattern of endothelial cell markers (CD93, CDH5, and PECAM1), and a leukocyte marker (PTPRC). Figure S2: genetic profile of EC subclusters (2). Figure S3: list of biocompare functional assay of EC subclusters (2). Figure S4: Expression of phagocytosis related genes in EC (ATP1B3) (2). [file 5412007.f1.zip › Supplementary Figure 4. Expression of phagocytosis related genes in EC(ATP1B3) (2).pdf]
